# Prevalence of osteoporosis and osteopenia among cancer patients and its risk factors: a retrospective monocentric study

**DOI:** 10.11604/pamj.2023.44.179.36365

**Published:** 2023-04-17

**Authors:** Ahmed Badheeb, Mohamed Al Sulieman, Faisal Ahmed, Ahmed Asiri, Mohamed Badheeb

**Affiliations:** 1 Department of Oncology, King Khalid Hospital, Najran, Saudi Arabia,; 2 Department of Medicine, Faculty of Medicine, Hadhramaut University, Hadhramaut, Yemen,; 3 Department of Internal Medicine, King Khalid Hospital, Najran, Saudi Arabia,; 4 Urology Research Center, Al-Thora General Hospital, Department of Urology, School of Medicine, Ibb University of Medical Sciences, Ibb, Yemen,; 5 Department of General Medicine, King Khalid Hospital, Najran, Saudi Arabia

**Keywords:** Osteoporosis, osteopenia, cancer, chemotherapy, hormone therapy

## To the editors of the Pan African Medical Journal

Decreased bone mineral density (BMD) is attributed to reducing bone mass index with architectural distortion, resulting in fractures [1]. Following adjuvant chemotherapy, cancer patients are more likely to develop osteopenia and osteoporosis, which affects two-thirds of males, more than half of premenopausal women, and approximately one-fifth of postmenopausal women [2]. Cancer therapy-induced bone loss is a leading cause of secondary osteoporosis, which results in bone fragility, increased fracture risk, decreased quality of life, and increased mortality [2]. Recent clinical guidelines recommend evaluating BMD in high-risk patients [3]. In Saudi Arabia, published data scarce on the impact of chemotherapy on bone loss among cancer survivors in the literature [4]. We report the prevalence of osteoporosis and osteopenia and their associated factors in cancer patients managed within the cancer center of King Khalid Hospital in Najran, Saudi Arabia between February 2021 and March 2022. 39 adult chemotherapy cancer, were interviewed. Using a Dual-energy X-ray absorptiometry (DXA) scan, the BMD of the lumbar spine and femur neck were evaluated. Univariate analysis was performed to determine the risk factors of osteoporosis and osteopenia with other variables. The patients' mean age was 60.15 ± 12.26 years. The majority of patients (53.8%) were aged between (60-69) years old, and most of them (64.1%) were female. The main primary diagnosis was breast cancer in 14 (35.9%) patients. Osteopenia, osteoporosis, and normal BMD were found in 7(17.9%), 28(71.8%), and 4 (10.3%) patients, respectively. Most of the patients (43.6%) had a low serum concentration of 25-hydroxycholecalciferol (25-vitamin D) ≤ 20 (ng/ml). Univariate analysis showed no relation between osteopenia or osteoporosis with gender, type of cancer, hormone therapy, length of hormone therapy or chemotherapy, history of bone pain, and metastatic stage (p >0.05). Osteoporosis and osteopenia were significantly more frequent in older patients, patients receiving chemotherapy, and patients with lower serum levels of vitamin D (p = 0.04, 0.05, and 0.005, respectively) (Table 1).

**Table 1:** comparison between osteopenia, osteoporosis, and normal bone health status with other factors

Variables	Subgroup	Total N (%)	Osteopenia N (%) and Mean (SD) 7(17.9)	Osteoporosis N (%) and Mean (SD) 28(71.8)	Normal N (%) and Mean (SD) 4(10.3)	P-value*
Age (year)	-	60.15 ± 12.26	55.71 ± 4.11	62.29 ± 12.73	53.00 ± 16.00	0.213
Age group (year)	<60	12(30.8)	5(41.7)	6(50.0)	1(8.3)	0.044
	≥60	27(69.2)	2(7.4)	22(81.5)	3(11.1)	
Gender	Male	14 (35.9)	1(7.1)	10(71.4)	3(21.4)	0.139
	Female	25 (64.1)	6(24.0)	18(72.0)	1(4.0)	
Primary diagnosis	Breast cancer	14(35.9)	2(14.3)	11(78.6)	1(7.1)	0.218
	Prostate cancer	10(25.6)	1(10.0)	6(60.0)	3(30.0)	
	Other	15 (38.5)	4(26.7)	11(73.3)	0(0.0)	
History of bone pain	Yes	18(46.2)	2(11.1)	14(77.8)	2(11.1)	0.597
	No	21(53.8)	5(23.8)	14(66.7)	2(9.5)	
Chemotherapy use	Yes	26(66.7)	4(15.4)	21(80.8)	1(3.8)	0.05
	No	13(33.3)	3(23.1)	7(53.8)	3(23.1)	
Duration of chemotherapy	≥ 5years	9(23.1)	2(22.2)	6(66.7)	1(11.1)	0.522
	4-2 years	6(15.4)	1(16.7)	5(83.3)	0(0.0)	
	≤ 1 year	11(28.2)	1(9.1)	10(90.9)	0(0.0)	
Hormone therapy use	Yes	25(64.1)	4(16.0)	17(68.0)	4(16.0)	0.372
	No	14(35.9)	3(21.4)	11(78.6)	0(0.0)	
Duration of hormone therapy	≥ 5years	4(10.3)	1(25.0)	2(50.0)	1(25.0)	0.268
	4-2 years	12(30.8)	2(16.7)	7(58.3)	3(25.0)	
	≤ 1 year	9(23.1)	1(11.1)	8(88.9)	0(0.0)	
Serum Vit D level	<20 (ng/ml)	17(43.6)	1(5.9)	16(94.1)	0(0.0)	0.005
	20-30 (ng/ml)	10(25.6)	4(40.0)	6(60.0)	0(0.0)	
	≥ 30 (ng/ml)	12(30.8)	2(16.7)	6(50.0)	4(33.3)	
Bone scan	No bone metastasis	24(61.5)	4(16.7)	17(70.8)	3(12.5)	1.000
	Bone metastasis	15(38.5)	3(20.0)	11(73.3)	1(6.7)	
Aware of jaw necrosis	Yes	10(25.6)	3(30.0)	7(70.0)	0(0.0)	0.292
	No	29(74.4)	4(13.8)	21(72.4)	4(13.8)	

* P-values < 0.05 were considered significant

Al Amri *et al*. reported a higher trend of osteopenia and osteoporosis in patients aged 50 years old or younger [4]. Another study found a higher prevalence of osteoporosis among older cancer survivors, as seen in our patients [5]. Various factors that may influence BMD in cancer patients were reported in different studies. For instance, in Reuss-Borst's study, age, body weight, menopausal state, and hormonal replacement therapy (HRT) in women and body weight in men have shown a significant association with the prevalence of osteoporosis [6]. Choi *et al*. showed a higher prevalence of osteoporosis among older age, female gender, and lower monthly income cancer survivors. Additionally, being underweight and inadequate calcium consumption in male cancer survivors were associated with osteoporosis [5]. In our analysis, older age, chemotherapy use, and low level of vitamin D were significantly associated with the prevalence of osteoporosis and osteopenia. However, other risk factors, including hormone therapy, duration of chemotherapy, and metastatic status, were not statistically significant. We explained that the small sample size and the short follow-up period limited us from making a robust statistical analysis. Therefore, a multicentric study is recommended.

Chemotherapy-associated bone loss in premenopausal women may be due to premature ovarian failure or, possibly, through estrogen-independent mechanisms. However, the more substantial bone loss was attributed to hormonal therapy due to its direct impact on osteogenesis and a longer administration time. Findings that are consistent with our results [7]. Pharmacological interventions for bone loss are recommended for patients with high risk and insufficient dietary intake, which include vitamin D supplementation (1000-2000 IU daily) and calcium supplementation (1000 mg daily) [8]. The addition of antiresorptive therapy should be recommended for patients with a baseline T score of -2.0 or patients with two or more clinical risk factors for fracture [9]. In our patients, instead of vitamin D, denosumab and calcium were administrated to all patients.

The current study had several limitations. Firstly, a retrospective nature, monocentric, and a small number of participants limited us from making a robust statistical analysis. Second, the incidence of osteopenia and osteoporosis was determined based DEXA scan and may be subject to misclassification. Finally, factors such as physical activity, economic status, comorbidity conditions, and diet, which may influence osteopenia and osteoporosis status, are not included.

## Conclusion

Bone loss due to cancer treatment is a significant potential adverse side effect. Those at risk of treatment-related bone loss should be identified using effective screening procedures, and therapy should be offered if risk factors are found. Our study found that osteoporosis and osteopenia were more common in older patients, patients under chemotherapy, and those with low levels of serum vitamin D.
